# Our Journal for 1885

**Published:** 1884-12-20

**Authors:** 


					﻿EDITORIALS AND MISCELLANEOUS.
Our Journal for 1885.—Our Journal for 1885 will in no respect fall be-
hind its former excellency, but will be more than ever adapted to the wants of
the busy practitioner. We shall endeavor to make the Record a practical,
plain and useful journal, and as such we doubt not our journal will continue
to grow and to extend its circulation. We trust that our readers will renew
promptly for 1885. Remember our offer to send the Journal for $1.00 to any
subscriber who will send with his renewal $2.00 and a new subscriber; or for
$3.00 will send the journal to himself and the friend whom he induces to sub-
scribe for 1885.
				

